# Triglyceride-glucose index is capable of identifying metabolically obese, normal-weight older individuals

**DOI:** 10.1186/s40101-024-00355-6

**Published:** 2024-02-03

**Authors:** Bokun Kim, Keisuke Taniguchi, Tomonori Isobe, Sechang Oh

**Affiliations:** 1 Future Convergence Research Institute, Changwon National University, Changwon, Republic of Korea; 2 Human Community Renovation Research Center, R Professional University of Rehabilitation, Tsuchiura, Ibaraki Japan; 3 Faculty of Rehabilitation, R Professional University of Rehabilitation, 2-10-35 Kohoku, Tsuchiura, Ibaraki 300-0032 Japan; 4 Faculty of Medicine, University of Tsukuba, 1-1-1 Tennodai, Tsukuba, Ibaraki 305-8575 Japan

**Keywords:** Chronic disease, Geriatric population, Metabolically obese but normal weight, Obesity, Triglyceride-glucose index

## Abstract

**Background:**

The concept of metabolically obese, normal weight (MONW) has emerged to describe individuals with a normal body mass index (BMI) who are at a relatively high risk of chronic diseases. However, BMI itself is a suboptimal index for the assessment of the health risks associated with visceral fat. The triglyceride-glucose (TyG) index is considered to be a reliable and cost-effective marker of insulin resistance. Therefore, in the present study, we aimed to determine the TyG index cut-off values that could be used to define MONW in older people and to determine the usefulness of these values for the prediction of chronic diseases.

**Methods:**

A total of 4,721 participants in the Korea National Health and Nutritional Examination Survey who were ≥ 60 years of age and did not have underweight or obesity were included. MONW was defined using the criteria for metabolic syndrome (MS), and the TyG index was calculated on the basis of the fasting plasma triglyceride and glucose concentrations. Chronic diseases, including T2DM, hypertension, and non-alcoholic fatty liver disease (NAFLD), were diagnosed.

**Results:**

The prevalence of MS increased from the lowest to the highest TyG index tertile. The cut-off values of the TyG index for MONW were calculated as 8.88 and 8.80 for males and females, respectively. MONW, defined using these cut-off values, was associated with high odds ratios for NAFLD, T2DM, and hypertension in both males and females.

**Conclusions:**

The TyG index cut-off values calculated in the present study can be used to discriminate individuals with MONW from other older individuals without obesity and to predict the risk of chronic diseases. These findings show that the TyG index is an effective and cost-efficient method of assessing the risk of chronic diseases in people with MONW.

**Supplementary Information:**

The online version contains supplementary material available at 10.1186/s40101-024-00355-6.

## Background

The rapid increase in the prevalence of obesity worldwide is a major contributor to the rising prevalence of several chronic non-communicable diseases, including type 2 diabetes mellitus (T2DM), hypertension, and non-alcoholic fatty liver disease (NAFLD), and therefore contributes to an enormous socioeconomic burden worldwide [1–3]. For decades, body mass index (BMI) has been widely used to quantify the level of obesity [4–6]. However, the limitations of its use have become increasingly apparent. BMI is a measure of body weight relative to height, but it cannot discriminate visceral fat, which predisposes toward several chronic diseases, and therefore a BMI-based assessment of obesity cannot fully explain the prevalence of such conditions [4–6].

Another group of people, “metabolically obese, normal weight (MONW)” individuals, have been shown to be of significant public health concern. MONW individuals are characterized by insulin resistance and are highly susceptible to chronic diseases, despite having BMIs < 25 kg/m^2^ [7–9]. In particular, there are significant numbers of older Asian people who do not have obesity and Asian MONW adults with T2DM, cardiovascular disease (CVD), or other conditions [9, 10]. The incidences of CVD and all-cause mortality for these people are even higher than those for their metabolically healthy counterparts who have obesity [9]. These findings highlight the need for an accurate marker of MONW, especially for older Asian people who do not have obesity, in order to prevent or manage the progression of diseases involving metabolic abnormalities.

In recent years, the triglyceride-glucose index (TyG index) has been shown to be a valid and reliable index for the assessment of insulin resistance; it was found to be significantly associated with hyperinsulinemic-euglycemic clamp data collected in Korea, Mexico, and Brazil [11–13] and it outperforms the homeostasis model of assessment-insulin resistance (HOMA-IR) for the identification of several chronic diseases associated with insulin resistance [14, 15]. In addition, the TyG index is a more cost-effective way of assessing insulin resistance than the two other methods named above because the plasma triglyceride and glucose concentrations are typically measured during health check-ups in many countries, such as Korea and Japan. Therefore, the authors hypothesized that the TyG index could be used to discriminate individuals with MONW from others in the older Korean population.

To test this hypothesis, we conducted a population-based cross-sectional study to determine the cut-off values of the TyG index that could be used to discriminate individuals who are MONW among older Korean people. In addition, we aimed to determine the usefulness of these TyG index cut-off values for MONW for the prediction of the risks of T2DM, hypertension, and NAFLD in this population.

## Materials and methods

### Study design and participants

**Fig. 1:**
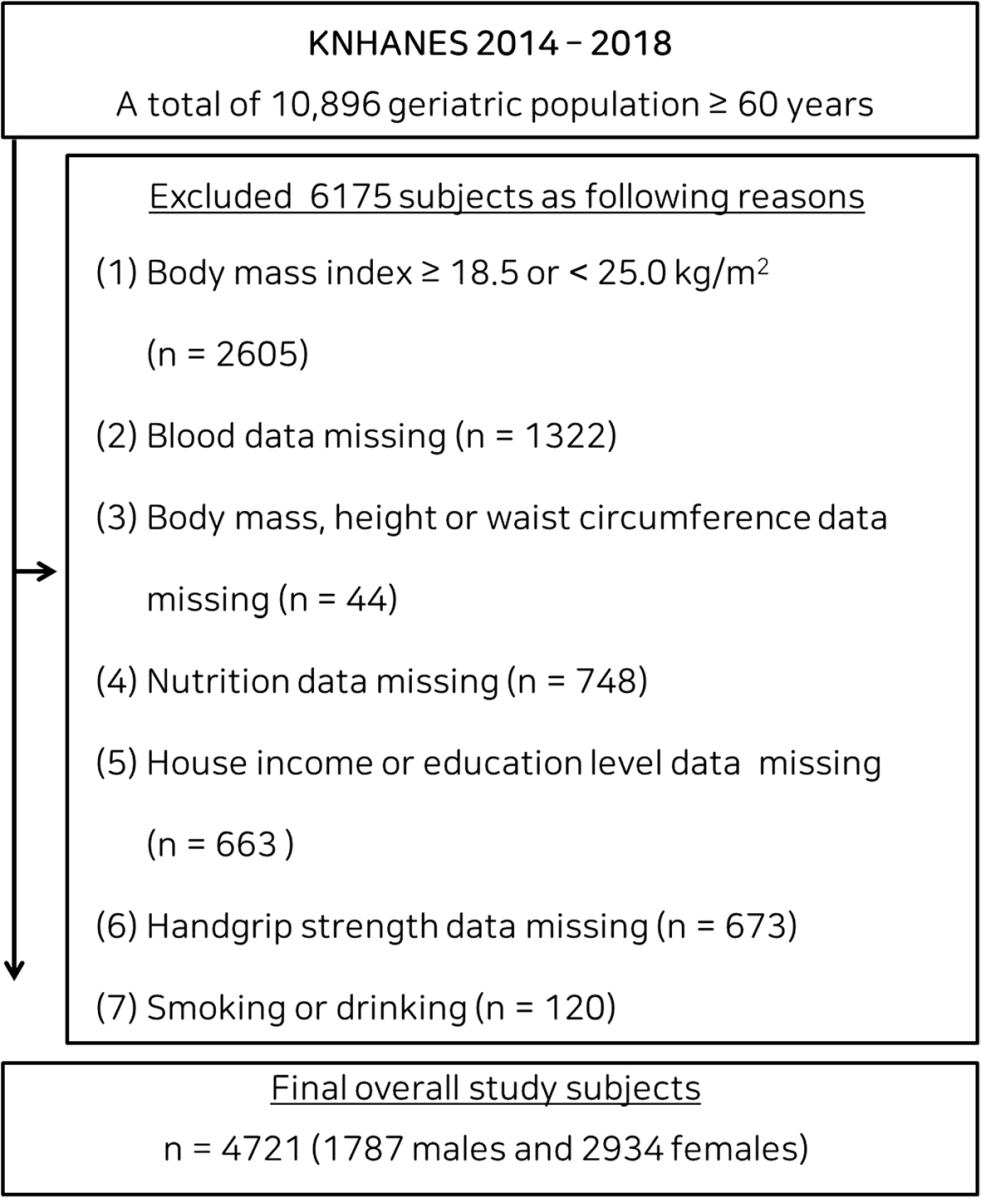
Flow diagram describing the classification of the data for the participants

For this study, we used a database containing data regarding the general health and nutritional status and lifestyle of South Koreans participating in the Korea National Health and Nutritional Examination Survey (KNHANES) that were collected between 2014 and 2018. Established in 1984, KNHANES is an ongoing national surveillance system for the assessment of the health and nutritional status of inhabitants of South Korea [16]. A total of 4,721 participants (1,787 males and 2,934 females) were selected from among all the participants ≥ 60 years of age who were not underweight and did not have obesity (BMI ≥ 18.5 and < 25.0 kg/m^2^) in KNHANES 2014–2018 (Fig. 1). Each participant provided written informed consent, and the study was conducted in accordance with the principles of the Declaration of Helsinki and approved by the Institutional Review Board of the University of Silla. (approval number 1041449–202203-HR-001).

### Measurements made

The height of the participants was measured to the nearest 0.1 cm while they were standing and not wearing shoes, and their body weight was measured to the nearest 0.1 kg using digital electronic scales while they were wearing light clothing. BMI was calculated as body weight divided by height squared (kg/m^2^). Waist circumference (WC) was measured to the nearest 0.1 cm using a glass fiberoptic tape measure. The blood pressure of the participants was measured manually three times in a mobile health monitoring vehicle and the mean values were used in the analyses. Blood samples were taken in the morning after an overnight fast of > 8 h. The circulating concentrations of glucose, triglycerides, and high-density lipoprotein (HDL)-cholesterol were determined using enzymatic or homogeneous enzymatic colorimetric methods, using a Hitachi 7600–210 automatic analyzer (Tokyo, Japan). HbA1c was determined by high-performance liquid chromatography using a Tosoh G8 (Tosoh, Tokyo, Japan).

### Assessment of MONW, MHNW, TyG index, and chronic diseases

MONW and metabolically healthy, normal-weight (MHNW) participants were defined as those with and without metabolic syndrome (MS), respectively, because MS is a constellation of metabolic abnormalities used to define MONW in previous studies. MS was diagnosed using the modified National Cholesterol Education Project Adult Treatment Panel (NCEP ATP III) criteria [17], which require the presence of three or more of the following components: (1) WC ≥ 90 cm in males and ≥ 80 cm in females; (2) systolic blood pressure (SBP) ≥ 130 mmHg, diastolic blood pressure (DBP) ≥ 85 mmHg, or use of anti-hypertensive medication; (3) fasting plasma triglyceride concentration > 150 mg/dL or the use of lipid-lowering medication; (4) fasting HDL-cholesterol concentration < 40 mg/dL in males and < 50 mg/dL in females, or the use of lipid-lowering medication; and (5) fasting plasma glucose concentration ≥ 100 mg/dL or the use of anti-hyperglycemic medication. The TyG index was calculated as ln [triglycerides concentration (mg/dL) × fasting plasma glucose concentration (mg/dL)]/2 [12, 16]. NAFLD was diagnosed using a hepatic steatosis index (HSI) > 36, according to the previously validated prediction model, and calculated as 8 × plasma alanine aminotransferase (ALT) activity [U/L]/ plasma aspartate aminotransferase (AST) activity [U/L] + BMI [+ 2 if diabetes mellitus is present; + 2 if female] [18, 19]. T2DM was diagnosed using an HbA1c ≥ 6.5% or the use of anti-hyperglycemic medication. The definition of hypertension used was that described above.

### Statistical analysis

Data are presented as mean ± standard deviation (SD). The independent *t*-test or the Mann–Whitney U test was used to compare datasets in males and females. One-way ANOVA was used to compare the sex-specific characteristics of the three groups, and the Bonferroni *post-hoc* test was used when ANOVA indicated an overall significant difference (*P* < 0.05). The Mann–Whitney U test was used to analyze group differences in the presence of a non-normal data distribution (*P* < 0.05). The Jonckheere-Terpstra test was used to detect trends in data across the three groups (two-tailed, *P* < 0.05). The sex-specific distributions and trends of MS and its components, according to TyG Index tertile, were explored using chi-square and linear-by-linear association. The optimal cut-off values of the TyG index for the identification of MONW in males and females were derived from receiver operating characteristic (ROC) curves. The area under the ROC curve (AUC), sensitivity, and specificity of each were calculated. This analysis was performed using MedCalc for Windows ver. 9.1.0.1 (MedCalc® Corp, Mariakerke Ostend, Belgium). *P* < 0.05 was accepted as indicating statistical significance. Logistic regression was used to evaluate the sex-specific relationships between MONW and chronic diseases. The fully adjusted model was adjusted for the potential confounders of age, educational level, household income, smoking, alcohol consumption, hand strength, moderate-to-vigorous physical activity, and diet, which are known or suspected to affect the relationships with chronic diseases. Statistical analyses were performed using SPSS software, ver. 26.0 (IBM, Inc., Armonk, NY, USA), with the exception of the ROC curve analysis.

## Results

Table 1 shows the characteristics of the participants. The mean age and BMI of the participants as a whole were 64.4 (SD 12.0) years and 22.7 (1.4) kg/m^2^, respectively, and the mean age and BMI of the male participants were significantly higher than those of the females (*P* < 0.001). The mean TyG index was 8.6 for both males and females, with standard deviations of 0.5 and 0.6, respectively, and no significant difference between the two groups. Additional information regarding the participants can be found in the Supplementary material (Table S1).

**Table 1:** Characteristics of the participants

	Overall (*n* = 4721)	Male (*n* = 1787)	Female (*n* = 2934)	*P* Value
Age, year^a^	64.4 ± 12.0	69.9 ± 6.3	61.1 ± 13.3	< 0.001
TyG Index	8.6 ± 0.6	8.6 ± 0.5	8.6 ± 0.6	= 0.201
Height, cm^a^	159.3 ± 8.1	166.0 ± 5.8	155.2 ± 6.4	< 0.001
Body weight, kg^a^	57.7 ± 6.8	62.9 ± 5.8	54.5 ± 5.3	< 0.001
Body mass index, kg/m^2a^	22.7 ± 1.4	22.8 ± 1.3	22.6 ± 1.4	< 0.001
Waist circumference, cm^a^	80.6 ± 6.5	84.3 ± 5.6	78.3 ± 6.0	< 0.001
SBP, mm Hg	119.7 ± 16.7	119.9 ± 16.6	119.6 ± 16.8	= 0.450
DBP, mm Hg	75.4 ± 10.0	75.6 ± 9.9	75.3 ± 10.0	= 0.329
FPG, mg/dL	99.9 ± 19.2	99.9 ± 19.0	99.9 ± 19.3	= 0.965
HDLC, mg/dL	51.1 ± 12.6	51.0 ± 12.3	51.1 ± 12.8	= 0.747
Triglyceride, mg/dL	124.3 ± 67.4	125.3 ± 66.1	123.8 ± 68.2	= 0.453
Medication				
Antihypertensive medication, n (%)	1611 (34.1)	725 (40.6)	886 (30.2)	< 0.001
Lipid-lowering medication, n (%)	832 (17.6)	267 (14.9)	565 (19.3)	< 0.001
Antihyperglycemic medication, n (%)	664 (14.1)	328 (18.4)	336 (11.5)	< 0.001
Chronic disease prevalence				
Non-alcoholic fatty liver disease, n (%)	415 (8.8)	98 (5.5)	317 (10.8)	< 0.001
T2DM, n (%)	1053 (22.3)	473 (26.5)	580 (19.8)	< 0.001
Hypertension, n (%)	2578 (54.6)	1078 (60.3)	1500 (51.1)	< 0.001

Values are mean ± SD

*Abbreviations*: *DBP* diastolic blood pressure, *FPG* fasting plasma glucose, *HDLC* high-density lipoprotein-cholesterol, *SBP* systolic blood pressure, *TyG* triglyceride-glucose, *T2DM* type 2 diabetes mellitus

^a^The Mann–Whitney U-test was used to assess the difference between groups

Table 2 shows the sex-specific characteristics and trends in the anthropometric and MS components, according to tertile of TyG index. In both male and female participants, we observed significant increasing trends in SBP and DBP from the lowest to the highest tertile, in contrast to the decreasing trend observed in the HDL-cholesterol concentration (*P* < 0.001 for all). In addition, post-hoc testing showed significant differences among the three tertiles with respect to SBP, DBP, and HDL-cholesterol concentrations. Specifically, there were increases in SBP and DBP, and a decrease in HDL-cholesterol, across the lowest, middle, and highest tertiles. Table S2 shows the results of the analyses of the other parameters and several covariates in males and females.

**Table 2:** Sex-specific characteristics and trends in anthropometric and metabolic syndrome components, according to TyG index tertile

	L (7.86 ± 0.22)	M (8.77 ± 0.12)	H (9.32 ± 0.25)	Post-hoc	Trend^b^
**Male (*n* = 1787)**	*n* = 604	*n* = 590	*n* = 593		
Age, year	69.8 ± 6.2	69.8 ± 6.3	70.0 ± 6.3	ns	= 0.564
Height, cm	166.0 ± 5.8	165.9 ± 5.8	165.9 ± 5.7	ns	= 0.660
Body weight, kg	63.1 ± 5.7	62.8 ± 5.9	62.9 ± 5.9	ns	= 0.756
Body mass index, kg/m^2^	22.9 ± 1.3	22.8 ± 1.3	22.8 ± 1.4	ns	= 0.553
Waist circumference, cm	84.5 ± 5.6	84.2 ± 5.3	84.3 ± 5.8	ns	= 0.625
Triglyceride, mg/dL^a^	67.0 ± 15.1	111.8 ± 20.9	198.0 ± 59.7	L < M < H	< 0.001
HDLC, mg/dL^a^	57.3 ± 12.6	51.1 ± 10.9	44.6 ± 9.6	L > M > H	< 0.001
FPG, mg/dL^a^	92.0 ± 9.9	99.3 ± 15.8	108.6 ± 24.5	L < M < H	< 0.001
SBP, mm Hg	115.4 ± 16.1	119.9 ± 16.4	124.5 ± 15.9	L < M < H	< 0.001
DBP, mm Hg^a^	73.6 ± 9.0	75.3 ± 9.7	77.9 ± 10.6	L < M < H	< 0.001
**Female (*n* = 2934)**	*n* = 973	*n* = 988	*n* = 973		
Age, year^a^	60.4 ± 13.5	61.4 ± 12.9	61.5 ± 13.6	ns	= 0.138
Height, cm	155.3 ± 6.5	155.4 ± 6.5	155.0 ± 6.3	ns	= 0.256
Body weight, kg	54.6 ± 5.2	54.6 ± 5.3	54.4 ± 5.4	ns	= 0.399
Body mass index, kg/m^2a^	22.6 ± 1.4	22.6 ± 1.3	22.6 ± 1.4	ns	= 0.802
Waist circumference, cm	78.2 ± 6.0	78.5 ± 6.0	78.1 ± 6.0	ns	= 0.581
Triglyceride, mg/dL^a^	63.8 ± 15.0	110.2 ± 19.4	197.5 ± 64.7	L < M < H	< 0.001
HDLC, mg/dL^a^	58.0 ± 13.0	51.1 ± 11.4	44.4 ± 9.9	L > M > H	< 0.001
FPG, mg/dL^a^	91.8 ± 8.9	98.5 ± 14.2	109.4 ± 26.1	L < M < H	< 0.001
SBP, mm Hg	113.6 ± 15.9	120.6 ± 16.6	124.6 ± 16.0	L < M < H	< 0.001
DBP, mm Hg^a^	72.8 ± 9.3	75.1 ± 9.8	77.9 ± 10.2	L < M < H	< 0.001

Values are mean ± SD

*Abbreviations*: *DBP* diastolic blood pressure, *FPG* fasting plasma glucose, *H* highest tertile, *HDLC* high-density lipoprotein-cholesterol, *L* lowest tertile, *M* middle tertile, *ns* not significant, *SBP* systolic blood pressure, *TyG* triglyceride-glucose

^a^The Mann–Whitney U-test was used to assess the difference between groups

^b^The Jonckheere-Terpstra test was used to assess the trend across the three groups

**Fig. 2:**
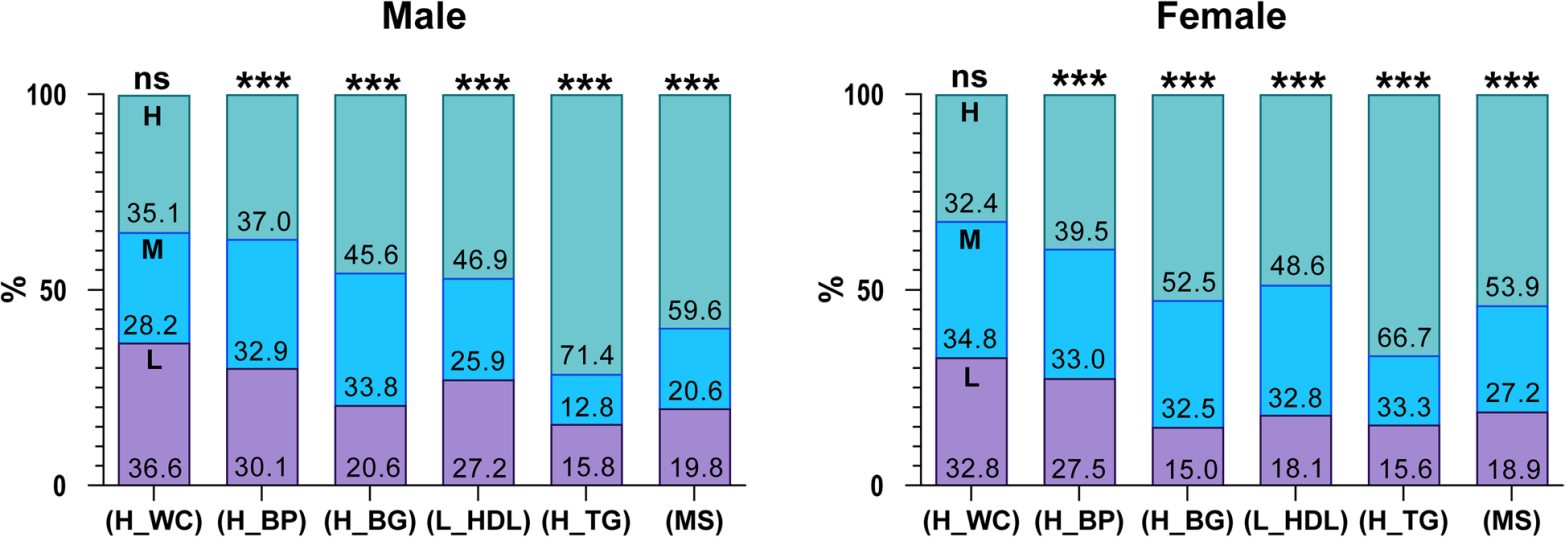
Sex-specific prevalence of metabolic syndrome and its components, according to tertile of TyG index. The sex-specific trend of MS prevalence and its components, according to TyG Index tertile, were analyzed using Jonckheere-Terpstra test. ***P < 0.001. Abbreviations: H, highest tertile; H_BP, hypertension or the use of antihypertensive medication; H_BG, hyperglycemia or the use of antihyperglycemic medication; H_WS, large waist circumference; H_TG, hypertriglyceridemia or the use of lipid-lowering medication; L, lowest tertile; L_HDL, low high-density lipoprotein-cholesterol concentration or the use of lipid-lowering medication; M, middle tertile; MS, metabolic syndrome; ns, not significant

The sex-specific prevalence of MS and its components, according to TyG Index tertile, is shown in Fig. 2. In both males and females, the prevalence of MS increased significantly from the lowest to the highest tertile (*P* < 0.001 for both). The percentage of participants with high triglyceride and glucose concentrations, high blood pressure or anti-hypertensive medication use, and low HDL-cholesterol concentration or lipid-lowering medication use showed significant increases from the lowest to the highest tertile (*P* < 0.001 for all).

**Fig. 3:**
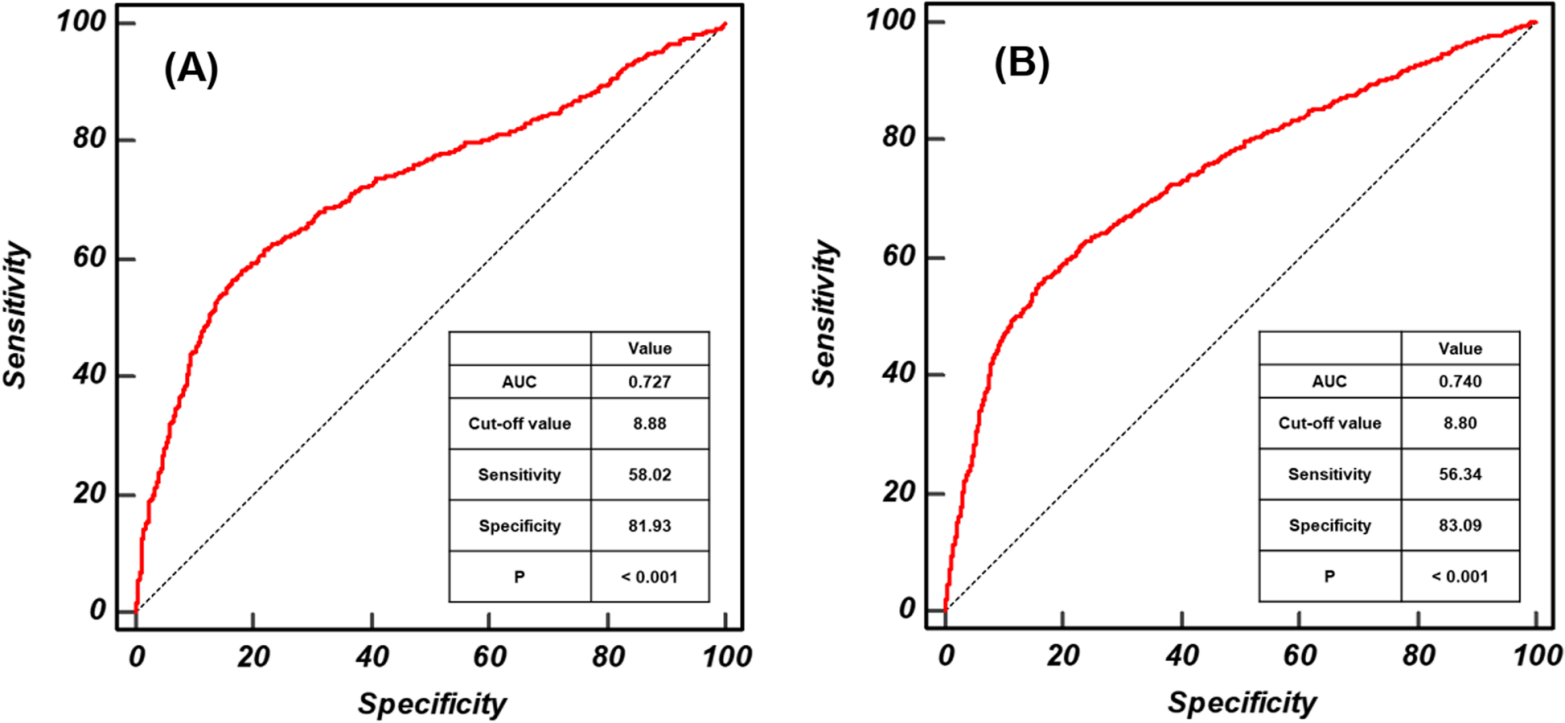
ROC curves for the TyG index in males and females with metabolic syndrome. The optimal cut-off values of the TyG index for the identification of MONW in males (**A**) and females (**B**) were derived from receiver operating characteristic (ROC) curves. Dotted line: reference; solid red line: AUC, indicating the accuracy of the TyG index for the identification of MS; cut-off value: the value of TyG index that predicts MS; sensitivity: the probability of individuals who had MS to be predicted to have MS; specificity: the probability of individuals who did not have MS to be predicted not to have MS. Abbreviations: AUC, area under the curve; MS, metabolic syndrome; TyG, triglyceride-glucose index

Figure 3 shows the ROC curves for the TyG index in males and females with MS. The highest sensitivity and specificity values were achieved with TyG indexes 8.88 (AUC: 0.727, sensitivity: 58.02%, and specificity: 81.93%) and 8.80 (AUC: 0.740, sensitivity: 56.34%, and specificity 83.09%) for males and females, respectively (*P* < 0.001 for both).

The sex-specific odds ratios for the relationships between MONW and chronic diseases are shown in Table 3. For males, in the unadjusted model, and compared with those who were MHNW, those who were MONW had odds ratios of 3.439 (95% confidence interval (CI): 2.344–5.044), 1.810 (1.454–2.254), and 1.613 (1.307–1.990) for NAFLD, T2DM, and hypertension, respectively. In the fully adjusted model, compared with participants who were MHNW, those who were MONW had odds ratios of 3.260 (2.203–4.826), 1.887 (1.508–2.361), and 1.591 (1.285–1.970) for NAFLD, T2DM, and hypertension, respectively. For females, in the unadjusted model, compared with participants who were MHNW, those who were MONW had odds ratios of 3.452 (95% CI: 2.770–4.301), 2.203 (1.831–2.649), and 1.812 (1.553–2.115) for NAFLD, T2DM, and hypertension, respectively. In the fully adjusted model, compared with participants who were MHNW, those who were MONW had odds ratios of 3.184 (2.532–4.004), 2.271 (1.871–2.758), and 1.845 (1.556–2.187) for NAFLD, T2DM, and hypertension, respectively.

**Table 3:** Sex-specific odds ratios for the relationships between the TyG index cut-off values and chronic diseases

	Unadjusted model	Fully adjusted model
**Male**		
MONW (*n* = 562)		
NAFLD	3.439 (2.344 - 5.044)^***^	3.260 (2.203 - 4.826)^***^
T2DM	1.810 (1.454 - 2.254)^***^	1.887 (1.508 - 2.361)^***^
Hypertension	1.613 (1.307 - 1.990)^***^	1.591 (1.285 - 1.970)^***^
MHNW (*n* = 1225)	reference	reference
**Female**		
MONW (*n* = 1025)		
NAFLD	3.452 (2.770 - 4.301)^***^	3.184 (2.532 - 4.004)^***^
T2DM	2.203 (1.831 - 2.649)^***^	2.271 (1.871 - 2.758)^***^
Hypertension	1.812 (1.553 - 2.115)^***^	1.845 (1.556 - 2.187)^***^
MHNW (*n* = 1909)	reference	reference

Values are odds ratio (95% confidence interval). The fully adjusted model was adjusted for age, educational level, household income, smoking, alcohol consumption, hand-grip strength, moderate-to-vigorous physical activity, and nutrition

*Abbreviations*: *MHNW* metabolically healthy, normal weight, *MONW* metabolically obese, normal weight, *NAFLD* non-alcoholic fatty liver disease

^*^P < 0.05

^**^P < 0.01

^***^P < 0.001

## Discussion

The present population-based cross-sectional study was the first to determine sex- and age-specific TyG index cut-off values that are capable of discriminating individuals who are MONW. Moreover, the usefulness of these values for predicting the risk of chronic diseases in older Korean adults was explored. The following results were obtained. First, the cut-off values of the TyG index for the discrimination of individuals who are MONW were calculated as 8.88 and 8.80 for males and females, respectively. Second, males who were MONW were 3.260, 1.887, and 1.591 times more likely to have NAFLD, T2DM, and hypertension; and females who were MONW were 3.184, 2.271, and 1.845 times more likely to have these chronic diseases. These results suggest that the TyG index cut-off values calculated in the present study are capable of discriminating individuals with MONW in older people without underweight or obesity and predicting the risk of chronic diseases.

The concept of MONW was first proposed by Ruderman et al. in 1981 [20]. Subsequently, its negative consequences, such as carotid atherosclerosis and arterial stiffness, high levels of subclinical vascular inflammation and oxidative stress, and an undesirable adipokine profile, have been reported numerous times [21–24]. However, a standardized definition of MONW has yet to be established. In previous studies, MONW has been defined as follows: having MS, being in the highest quartile of or within a specific range of HOMA-IR values, having excess visceral or total adiposity, having a low glucose disposal rate, or having several cardiovascular risk factors [25]. The conditions used in previous studies are all underpinned by insulin resistance; therefore, we investigated the utility of the TyG index as an indicator of MONW.

In the present study, trend testing showed significant increasing trends in the prevalence of MS, high triglyceride concentration, high glucose concentration, and blood pressure; and a significant decreasing trend in HDL-cholesterol from the lowest to the highest tertile (*P* < 0.001 for all) in both males and females. However, no trend in WC was identified (Fig. 2). Similarly, trend testing showed significant increasing or decreasing trends in triglyceride, glucose, SBP, DBP, and HDL-cholesterol from the lowest to the highest tertile in both sexes (*P* < 0.001 for all), but not for WC (Table 2). These results demonstrate the close relationship between the TyG index and MONW in older people.

Analysis of the ROC curves showed that the optimal TyG index cut-off values for the prediction of MONW are 8.88 (AUC: 0.727, sensitivity: 58.02%, specificity: 81.93%) and 8.80 (AUC: 0.740, sensitivity: 56.34%, specificity: 83.09%) for males and females, respectively. Prior to the present study, Shin et al., who studied 4,415 Korean males and females aged ≥ 20 years, calculated a TyG index cut-off value of 8.81 (AUC: 0.894, sensitivity: 86.7%, specificity: 80.1%) for discriminating both males and females with insulin resistance [26]. In addition, Lee et al. showed that middle-aged Korean males and females with a TyG index ≥ 8.8 (AUC: 0.751) are at higher risk of T2DM than those with values below this cut-off, suggesting that it may be relevant to the development of insulin resistance [27]. Because these previous studies did not provide sex- or age-specific cut-off values for the TyG index, direct comparison of the present and previous studies is not possible. However, considering the cut-off values obtained in the present and previous studies, a TyG index of approximately 8.80–8.90 appears suitable for identifying insulin resistance in the Korean population.

Males who were MONW, according to the TyG index cut-off value of 8.88, were 3.260, 1.887, and 1.591 times more likely to have NAFLD, T2DM, and hypertension, respectively; and females who were MONW, according to the TyG index cut-off value of 8.80, were 3.184, 2.271, and 1.845 times more likely to have these chronic diseases, respectively, than healthy individuals who were not obese. These findings can be explained using those of previous studies. Regarding NAFLD, Feng et al. and Stefan et al. showed that individuals who are MONW store more fat in their liver and are therefore more likely to have NAFLD [7, 8]. Regarding T2DM, Conus et al. and Lacobini et al. showed that individuals who are MONW are more likely to develop T2DM, because insulin resistance and low insulin sensitivity are features of MONW [28, 29]. Regarding hypertension, Kim et al. and Oliveros et al. reported that an abnormal lipid profile, and especially high triglyceride and low HDL-cholesterol concentrations, are common in individuals who are MONW [30, 31]. Because an abnormal lipid profile negatively affects blood pressure, individuals who are MONW are more susceptible to hypertension. Taking all of these findings together, individuals who are MONW according to the TyG index cut-off value generated in the present study are more susceptible to chronic diseases. This indicates the usefulness of the TyG index cut-off value generated in the present study.

The present study had both strengths and limitations. Important potential covariates were adjusted for, including demographic parameters and lifestyle factors that may influence the relationship between MONW and chronic diseases. However, because of the cross-sectional nature of the study design, the conclusions drawn regarding the predictive ability of MONW discriminated using the TyG index cut-off for chronic disease remain tentative. Therefore, longitudinal studies should be performed to validate the conclusion of the present study. In addition, because all the participants were older Korean adults, whether the results of the present study can be extrapolated to individuals of other ethnicities or in other nations is unclear. Therefore, further research should be conducted in participants of different ethnicities to assess the utility of the TyG index cut-off value and the relationship between MONW and chronic diseases.

## Conclusions

The TyG index cut-off values generated in the present study are capable of discriminating individuals who are MONW from other older individuals without underweight or obesity, and predicting the risk of chronic diseases. The findings of this study confirm that the TyG index is a useful and cost-effective method of assessing the risk of chronic diseases in individuals who are MONW.

### Supplementary Information

**Additional file 1: Table S1.** Characteristics of study subjects. **Table S2.** Sex-specific characteristics of biochemical parameters and lifestyle and trends by TyG Index tertiles.

## Abbreviations

ALT: Alanine aminotransferase
AST: Aspartate aminotransferase
AUC: Area under the ROC curve
BMI: Body mass index
CVD: Cardiovascular disease
DBP: Diastolic blood pressure
HDL: High-density lipoprotein
HOMA-IR: Homeostasis model of assessment-insulin resistance
KNHANES: Korea National Health and Nutritional Examination Survey
MHNW: Metabolically healthy, normal-weight
MONW: Metabolically obese, normal weight
MS: Metabolic syndrome
NAFLD: Non-alcoholic fatty liver disease
ROC: Receiver operating characteristic
SBP: Systolic blood pressure
SD: Standard deviation
TyG: Index triglyceride-glucose index
WC: Waist circumference
