# Supplementary material for: Triglyceride-glucose index is capable of identifying metabolically obese, normal-weight older individuals
Source: J Physiol Anthropol. 2024 Feb 3;43:8. doi: 10.1186/s40101-024-00355-6 (PMC10837892; doi:10.1186/s40101-024-00355-6)
Supplement: Supplementary file 1 — Additional file 1: Table S1. Characteristics of study subjects. Table S2. Sex-specific characteristics of biochemical parameters and lifestyle and trends by TyG Index tertiles. [file 40101_2024_355_MOESM1_ESM.docx]

| **Table S1.** Characteristics of study subjects | | | | | | | | | | |
| --- | --- | --- | --- | --- | --- | --- | --- | --- | --- | --- |
|  | Overall  (n = 4721) | | | Male  (n = 1787) | | | Female  (n = 2934) | | | *P* Value |
| TC, mg/dL | 191.7 ± 36.8 | | | 192.7 ± 37.1 | | | 191.1 ± 36.6 | | | = 0.143 |
| Creatinine, mg/dL | 0.8 ± 0.3 | | | 0.9 ± 0.2 | | | 0.8 ± 0.3 | | | = 0.143 |
| AST, U/L | 22.7 ± 9.4 | | | 22.6 ± 8.9 | | | 22.7 ± 9.7 | | | = 0.918 |
| ALT, U/L | 21.0 ± 14.4 | | | 21.0 ± 14.2 | | | 21.0 ± 14.5 | | | = 0.889 |
| HbA1c, % | 5.7 ± 0.7 | | | 5.7 ± 0.7 | | | 5.7 ± 0.7 | | | = 0.262 |
| HGs, kg | 28.2 ± 9.7 | | | 28.3 ± 9.7 | | | 28.2 ± 9.7 | | | = 0.708 |
| MVPA, min^†^ | 85.6 ± 226.7 | | | 94.3 ± 256.8 | | | 80.2 ± 206.1 | | | = 0.481 |
| TEI, kcal/d | 1974.7 ± 867.2 | | | 1985.3 ± 855.1 | | | 1968.2 ± 874.5 | | | = 0.511 |
| Protein, g/d | 69.5 ± 37.8 | | | 69.7 ± 38.4 | | | 69.3 ± 37.5 | | | = 0.731 |
| Fat, g/d | 41.6 ± 32.6 | | | 41.5 ± 33.1 | | | 41.6 ± 32.2 | | | = 0.911 |
| Carbohydrate, g/d | 308.4 ± 129.6 | | | 311.6 ± 128.5 | | | 306.5 ± 130.2 | | | = 0.191 |
| Household income (%) |  |  |  |  |  |  |  |  |  | < 0.01 |
| Low | 1427 (30.2) | | | 576 (32.2) | | | 851 (29.0) | | |  |
| Lower-middle | 1279 (27.1) | | | 547 (30.6) | | | 732 (24.9) | | |  |
| Upper middle | 1038 (22.0) | | | 350 (19.6) | | | 688 (23.4) | | |  |
| High | 977 (20.7) | | | 314 (17.6) | | | 663 (22.6) | | |  |
| Education level (%) |  |  |  |  |  |  |  |  |  | < 0.001 |
| Primary school | 1865 (39.5) | | | 633 (35.4) | | | 1232 (42.0) | | |  |
| Middle school | 655 (13.9) | | | 339 (19.0) | | | 316 (10.8) | | |  |
| High school | 1154 (24.4) | | | 466 (26.1) | | | 688 (23.4) | | |  |
| College | 1047 (22.2) | | | 349 (19.5) | | | 698 (23.8) | | |  |
| Drinking (%) |  |  |  |  |  |  |  |  |  | < 0.001 |
| Never | 1766 (37.4) | | | 466 (26.1) | | | 1300 (44.3) | | |  |
| ≤ once a week | 2070 (43.8) | | | 672 (37.6) | | | 1398 (47.6) | | |  |
| 2-3 times/week | 521 (11.0) | | | 344 (19.3) | | | 177 (6.0) | | |  |
| ≥ 4 times/week | 364 (7.7) | | | 305 (17.1) | | | 59 (2.0) | | |  |
| Smoking (%) |  |  |  |  |  |  |  |  |  | < 0.001 |
| Never | 3099 (65.6) | | | 359 (20.1) | | | 2740 (93.4) | | |  |
| Former smoking | 1166 (24.7) | | | 1051 (58.8) | | | 115 (3.9) | | |  |
| Current smoking | 456 (9.7) | | | 377 (21.1) | | | 79 (2.7) | | |  |
| Values are means ± SD (%).^†^Mann-Whitney U test was applied to assess the difference between groups. ALT, alanine transaminase; AST, aspartate aminotransferase; HbA1c, hemoglobin A1c; HGs, Handgrip strength, MVPA, moderate to vigorous physical activity; TC, total cholesterol; TEI, total energy intake | | | | | | | | | | |

| **Table S2.** Sex-specific characteristics of biochemical parameters and lifestyle and trends by TyG Index tertiles | | | | | |
| --- | --- | --- | --- | --- | --- |
|  | L  (7.86 ± 0.22) | M  (8.77 ± 0.12) | H  (9.32 ± 0.25) | Post-hoc | trend^‡^ |
| *Male* | n = 604 | n = 590 | n = 593 |  |  |
| AST, U/L^†^ | 20.91 ± 7.892 | 22.44 ± 8.532 | 24.58 ± 9.678 | L < M < H | < 0.001 |
| ALT, U/L^†^ | 16.9 ± 11.1 | 20.6 ± 13.3 | 25.5 ± 25.5 | L < M < H | < 0.001 |
| HbA1c, %^†^ | 5.5 ± 0.4 | 5.7 ± 0.7 | 6.0 ± 0.9 | L < M < H | < 0.001 |
| TC, mg/dL^†^ | 184.1 ± 34.1 | 193.1 ± 36.0 | 200.9 ± 39.1 | L < M < H | < 0.001 |
| Cre, mg/dL^†^ | 0.82 ± 0.16 | 0.86 ± 0.18 | 0.88 ± 0.18 | L < M, H | < 0.001 |
| HGs, kg^†^ | 26.7 ± 8.3 | 28.7 ± 10.4 | 29.5 ± 10.1 | L < M, H | < 0.001 |
| MVPA, min | 105.2 ± 290.5 | 90.3 ± 225.5 | 87.3 ± 249.4 | ns | < 0.05 |
| TEI, kcal/d^†^ | 1906.7 ± 771.2 | 2053.6 ± 942.6 | 1997.6 ± 839.7 | ns | = 0.087 |
| Protein, g/d^†^ | 68.2 ± 35.4 | 71.5 ± 41.6 | 69.5 ± 38.0 | ns | = 0.828 |
| Fat, g/d | 43.2 ± 32.6 | 42.2 ± 35.0 | 39.1 ± 31.6 | ns | < 0.01 |
| Carbo, g/d | 297.1 ± 121.6 | 325.0 ± 138.4 | 313.1 ± 123.6 | L < M | < 0.01 |
| *Female* | n = 973 | n = 988 | n = 973 |  |  |
| AST, U/L^†^ | 20.6 ± 7.2 | 22.2 ± 8.3 | 25.1 ± 12.2 | L < M < H | < 0.001 |
| ALT, U/L^†^ | 16.7 ± 10.4 | 19.8 ± 11.3 | 26.6 ± 18.6 | L < M < H | < 0.001 |
| HbA1c, %^†^ | 5.5 ± 0.4 | 5.7 ± 0.6 | 6.0 ± 0.9 | L < M < H | < 0.001 |
| TCI, mg/dL^†^ | 181.2 ± 31.6 | 193.4 ± 36.2 | 199.3 ± 39.7 | L < M < H | < 0.001 |
| Cre, mg/dL | 0.80 ± 0.45 | 0.83 ± 0.28 | 0.88 ± 0.27 | L, M < H | < 0.001 |
| HGs, kg^†^ | 26.4 ± 8.5 | 27.8 ± 9.6 | 30.3 ± 10.5 | L < M < H | < 0.001 |
| MVPA, min | 79.3 ± 179.0 | 82.7 ± 239.2 | 78.6 ± 194.9 | ns | = 0.495 |
| TEI, kcal/d^†^ | 1890.2 ± 782.5 | 1941.9 ± 850.5 | 2073.0 ± 971.1 | L, M < H | < 0.001 |
| Protein, g/d^†^ | 67.5 ± 32.9 | 68.9 ± 37.7 | 71.5 ± 41.2 | ns | = 0.238 |
| Fat, g/d | 43.4 ± 31.9 | 40.8 ± 32.6 | 40.7 ± 32.2 | L > M, H | < 0.01 |
| Carbo, g/d^†^ | 293.3 ± 119.9 | 306.4 ± 124.2 | 319.9 ± 144.2 | L < M, H | < 0.001 |
| Values are means ± SD. †Mann-Whitney U test was applied to assess the difference between groups. ‡Jonckheere-Terpstra test was used to assess the trend among three groups. ALT, alanine transaminase; AST, aspartate aminotransferase; Carbo, carbohydrate; Cre, creatinine; eGFR, estimated glomerular filtration rate; H, The highest tertile; HbA1c, hemoglobin A1c; HGs, handgrip strength; L, The lowest tertile; M, The middle tertile; MVPA, moderate to vigorous physical activity; ns, not significant; TEI, total energy intake; TC, total cholesterol. | | | | | |
